# Ubc9 Mediates SUMO2/3 Modification to Regulate Meiosis in Male Mice

**DOI:** 10.3390/biomedicines14092129

**Published:** 2026-09-21

**Authors:** Peng Lv, Wenchao Xu, Bin Yang, Dengjianyi Xu, Tao Wang, Jihong Liu, Xiaming Liu

**Affiliations:** Department of Urology, Tongji Hospital, Tongji Medical College, Huazhong University of Science and Technology, Wuhan 430030, Chinabinyangfz@163.com (B.Y.); tjhwt@126.com (T.W.); jhliu@tjh.tjmu.edu.cn (J.L.)

**Keywords:** Ubc9, male infertility, meiosis, synaptonemal complex

## Abstract

**Objective**: SUMOylation is a crucial protein modification that regulates various physiological and pathological processes. Ubc9 serves as a key ubiquitin-conjugating enzyme in the process of SUMOylation. However, the role of Ubc9 has yet to be elucidated during spermatogenesis in mammals. **Methods**: We analyzed the expression profile of Ubc9 in spermatogenesis via quantitative polymerase chain reaction, Western blotting, and meiotic chromosome spread assays. Stra8-EGFPCre mice were used to construct a germ cell-specific conditional Ubc9 knockout mouse model to explore the effects of Ubc9 on male fertility and testicular development. Histological and immunofluorescence staining were performed to observe the development of germ cells and the synaptonemal complex. Moreover, proteomics analysis of the testes was used to detect changes in the control and Ubc9-deficient mice at postnatal day 8. **Results**: Our results revealed that Ubc9 was highly expressed in the early stage of meiosis. Ubc9 was extensively distributed in the nucleus during meiosis I. Conditional Ubc9 knockout mice with Stra8-EGFPCre resulted in atrophy and reduced volume and mass of testes and complete loss of male fertility. Histological and immunofluorescence staining of testes revealed that the number of germ cells was significantly reduced in the seminiferous tubules in conditional Ubc9 knockout mice at postnatal day 9. Meiotic chromosome spread assays revealed that the synaptonemal complex was abnormally developed and arrested in meiosis I. Moreover, immunofluorescence staining with SUMO1 and SUMO2/3 revealed that Ubc9 regulated the development of the synaptonemal complex mainly via SUMO2/3 rather than SUMO1. The results of the proteomics analysis revealed that the levels of proteins related to the synaptonemal complex, including SYCE1, SYCE2, TEX12, SYCP1, DMC1, and Hormad1, were markedly decreased with Ubc9 deficiency. **Conclusions**: Taken together, our study demonstrated that Ubc9 influenced the development of the synaptonemal complex during meiosis I and is essential for male fertility in mammals.

## 1. Introduction

The process of spermatogenesis requires precise spatial and temporal regulation involving transcription, post-transcriptional modifications, and post-translational modifications of multiple genes [1]. Spermatogenesis is a crucial biological process in the production of haploid germ cells, including the proliferation and differentiation of spermatogonia, the meiosis of spermatocytes, and the transformation of round spermatids into elongated spermatids. This intricate process is highly susceptible to environmental factors and genes, which can ultimately lead to male sterility [2,3]. Only 4% of infertile men are diagnosed with clear genetic abnormalities, including chromosomal defects, point mutations in single genes, copy number variations, and polygenic abnormalities [4,5]. In addition, in other studies, it has been discovered that post-translational modifications, including phosphorylation, acetylation, ubiquitination, N6-methyladenosine modification, and SUMOylation, are essential for spermatogenesis and other cellular processes [1,6,7,8,9]. Thus, elucidating the functions of key modifier genes is crucial for the in vitro culture of germ cells and assisted reproductive technology.

The SUMO-modified enzyme-cascade reaction is similar to the process of ubiquitination, both of which involve the gradual transfer of small molecule modifications to substrates by activating enzymes and conjugating enzymes and ligases. Ubiquitination and SUMOylation interactively modify the same protein, playing a synergistic role in regulating the functions of the protein [10]. SUMOylation covalently attaches SUMO to the lysine residues of proteins, a process mainly involving SAE1/2, Ubc9, SUMO, PIAS, and SENP [11]. The SUMO-modified proteins in yeast affect various cellular processes, including telomere maintenance, kinetochore function, nucleolar dynamics, DNA synthesis, DNA damage response, sister chromatid cohesion, and chromosome condensation [12,13]. In a recent study, 775 targets of SUMOylation were identified during yeast meiosis through proteomics. Execution-point analysis revealed different key points of SUMOylation, and it was determined that SUMOylation plays a role at the onset of the S phase, DNA double-strand break (DSB) formation, chromosome crossing over, and synapsis [14]. Recent evidence suggests that SUMOylation targets entire protein complexes rather than individual substrates, differing from the typical behavior of post-translational modifications that exhibit high substrate specificity [15,16,17]. In budding yeast, adhesion proteins, cohesin, telomere-binding proteins, and nucleolar proteins are modified as a group [18]. When the SUMO E3 ligase binds to the protein complex, the entire protein complex will initiate SUMOylation [11].

Ubc9, as the only ubiquitin-conjugating enzyme in the SUMOylation cascade, possesses a specific domain called the ubiquitin-coupled catalytic (UBC) domain, which consists of 150 to 200 amino acids [19,20]. In human cancers, Ubc9 impacts cell growth, cancer development, and drug responsiveness through its involvement in the cell cycle, proliferation, apoptosis, and DNA repair [21,22,23]. Research has demonstrated that inhibiting the expression of Ubc9 leads to mitotic arrest in yeast at the G2 phase or early M phase, resulting in the formation of single nuclei, excessive DNA replication, and aberrant pre-anaphase spindles in the blastospore [24]. Moreover, Ubc9 is primarily located on the XY body during pachytene and diplotene spermatocytes, in addition to the centromeres of metaphase spermatocytes in mouse testes [25,26]. To date, however, the function of Ubc9 in male mammalian reproduction remains unknown.

In this study, we discovered that Ubc9 is highly expressed in the early stage of male meiosis. To further explore the critical role of Ubc9 in meiosis, we constructed a differentiated spermatogonia cell-specific Ubc9 conditional knockout mouse model using Stra8-EGFPCre mice. In Ubc9 conditional knockout adult male mice, we observed testicular atrophy, a significant reduction in germ cells, and subsequent loss of male fertility. Moreover, germ cells were arrested at the late zygotene stage and early pachytene stage with severe abnormalities of the synaptonemal complex. Interestingly, our findings revealed that Ubc9 mainly regulated the formation of the synaptonemal complex through SUMO2/3 rather than SUMO1. In addition, proteomics analysis revealed that Ubc9 deficiency resulted in reduced expression of synaptonemal complex-related proteins, such as Syce1, Syce2, Tex12, Sycp1, and Hormad1. In this article, we emphasize the crucial role of Ubc9 in promoting the formation of the synaptonemal complex during meiosis.

## 2. Methods

### 2.1. Ubc9 Conditional Knockout Mice

The animal experiments performed in this study were approved by the Institutional Animal Care and Use Committee of Tongji Hospital, Tongji Medical College, Huazhong University of Science and Technology (IACUC Issue NO.: TJH-202212007). Ubc9^flox/+^ mice were kindly donated by Prof. Cong-yi Wang at the Center for Biomedical Research, Tongji Hospital, Tongji Medical College, Huazhong University of Science and Technology [27]. Stra8-EGFPcre (Cyagen Biosciences, Santa Clara, CA, USA, C001283) mice were purchased from Cyagen Biosciences. All mice used in the experiments shared a C57BL/6J genetic background. All experimental mice were bred in a specific pathogen-free facility with adequate temperature and humidity conditions. Stra8-EGFPcre mice were crossed with Ubc9^flox/flox^ mice to obtain Stra8-EGFPcre; Ubc9^flox/+^ mice. Thereafter, adult Stra8-EGFPcre; Ubc9^flox/+^ mice were bred with Ubc9^flox/flox^ to generate Stra8-EGFPcre; Ubc9^flox/Δ^ mice (Δ represents deleted, designed as SUcKO). All animal experiments were conducted in accordance with the Cartagena Protocol on Biosafety. Genomic DNA was obtained from postnatal day 5 mouse toes to determine mouse genotyping. The primer information is presented in Table S1.

### 2.2. Fertility Test

To assess the fertility of the SUcKO male mice, we performed a natural mating test. Four adult SUcKO and four adult control male mice were used to perform the fertility test. Each male was mated with two 2-month-old female mice for six months. Female mice were assessed for the number of offspring produced.

### 2.3. Histology Staining

Mouse testes were fixed with an improved Davidson’s solution (mixture consisting of 37% formaldehyde solution, glacial acetic acid, and anhydrous ethanol) and other tissues were fixed with 4% paraformaldehyde (Sigma, Burlington, MA, USA, P6148). Following dehydration and embedding in paraffin, tissues were sectioned at 5 μm thickness and placed on adhesive glass slides. After deparaffinization, the slides were stained with hematoxylin and eosin (HE) (Servicebio, Wuhan, China, G1005) or a periodic acid–Schiff (PAS) staining Kit (Beyotime Biotechnology, Shanghai, China, C0142S). The subsequent processes of HE staining and PAS staining were performed in complete accordance with the instructions of the respective products. If required, readers are advised to refer to the corresponding catalog numbers to obtain the instructions for the experimental procedure.

### 2.4. Immunofluorescence

Sections were boiled in citrate antigen retrieval solution or EDTA antigen retrieval solution under medium heat for 15 min in a microwave and then gradually cooled at room temperature. Slides were blocked with 5% donkey serum solution for 1 h at room temperature and incubated with primary antibodies diluted in 5% donkey serum solution at 4 °C overnight. After washing with phosphate-buffered saline (PBS), the slides were incubated with secondary antibodies diluted in 5% donkey serum solution for 2 h at room temperature. After washing with PBS three times, the sections were stained and mounted with Antifade Mounting Medium with DAPI (Beyotime Biotechnology, P0131) for DNA staining. Images were obtained with a Zeiss Axio image microsystem. The antibody information is presented in Table S2.

### 2.5. Meiotic Chromosome Spread Analysis

Fresh testes were obtained from postnatal day 16 and 21 mice. Dispersed seminiferous tubules were incubated with hypotonic extraction buffer for 30 min at room temperature. The germ cells were separated from the seminiferous tubules into a sucrose solution. Thereafter, the cell suspension was evenly spread on the adhesive slides. After being fixed with 1% paraformaldehyde containing 0.1% Triton-100, the slides were washed with 0.4% Photo-Flo (Kodak, Rochester, NY, USA, 1464510). The dry slides were used for immunofluorescence staining.

### 2.6. RNA Isolation and Quantitative Real-Time PCR

Total RNA was extracted from tissue samples using VeZol Reagent (Vazyme, Nanjing, China, R411). The resulting RNA was reverse-transcribed into cDNA using the HiScript II Reverse Transcriptase Kit (Vazyme, R222). Quantitative real-time PCR was subsequently performed on a Real-Time PCR System with ChamQ Universal SYBR qPCR Master Mix (Vazyme, Q711). The threshold cycle (Ct) values were recorded for each reaction, and the relative expression levels of target genes were calculated using the 2^−ΔΔCt^ method, with normalization to the endogenous reference genes Gapdh and Tubulin. All experiments were conducted with three replicates, with each condition run in triplicate to ensure statistical reliability. The primer sequences are listed in Table S1.

### 2.7. Western Blotting Analysis

The mouse tissues were lysed using RIPA buffer containing cocktail buffer on ice for 15 min, followed by centrifugation at 12,000× *g* at 4 °C. The protein concentration was determined using the Pierce BCA protein assay kit according to the manufacturer’s protocol. Total proteins were denatured, after which equal amounts of protein were separated via SDS-PAGE and transferred to PVDF membranes. After blocking with 5% skim milk in TBST buffer for 1 h at room temperature, the membrane was probed with primary antibodies at 4 °C overnight, followed by three TBST washes and incubation with secondary antibodies for 1 h at room temperature. The membranes were further incubated in Western ECL substrates, and data collection was conducted using a Bio-Rad (Hercules, CA, USA) ChemiDoc MP detector system. The antibody information is presented in Table S2.

### 2.8. Proteomic Assay

Proteins were extracted from the postnatal day 8 mouse testes using RIPA lysis buffer (Beyotime Biotechnology, P0013J) containing cocktail buffer. The lysates were clarified via centrifugation at 12,000× *g* at 4 °C. Protein supernatants were obtained to calculate protein concentrations and control the quality of samples by using BCA testing and SDS-PAGE. Dithiothreitol and tris-(2-carboxyethyl)-phosphine hydrochloride solutions were added to the protein sample to hydrolyze disulfide bonds and protect sulfhydryl residues, promoting the degradation of the protein’s secondary structure. A sufficient amount of trypsin was added to hydrolyze the proteins into appropriately sized peptide segments. Thereafter, the samples were placed in the chromatography column to remove salt ions. The peptide solution was analyzed using the Bruker timsTOF Pro2 mass spectrometer, combined with the 4D-DIA method for differential proteomics analysis, including retention time, ion intensity, ion mobility, and mass-to-charge ratio. Data normalization and database comparison were performed, followed by qualitative and quantitative analyses. Proteins with a 1.5-fold change and a *p*-value (using Student’s *t*-test) less than 0.05 are defined as differentially expressed proteins. We defined proteins with an expression difference greater than 1.5 times and an inter-group *p*-value less than 0.05 as differentially expressed proteins.

### 2.9. Statistical Analysis

Quantitative results are presented as means ± standard deviation (SD) based on at least three independent samples. Statistical analyses were performed using GraphPad Prism 8.4 software and Excel software. The data were considered significantly different when the *p*-value was less than 0.05. *p*-values are shown in the figures and figure legends: * *p* < 0.05, ** *p* < 0.01, and *** *p* < 0.001.

## 3. Results

### 3.1. Ubc9 Is Highly Expressed in Mouse Germ Cells

**Figure 1:**
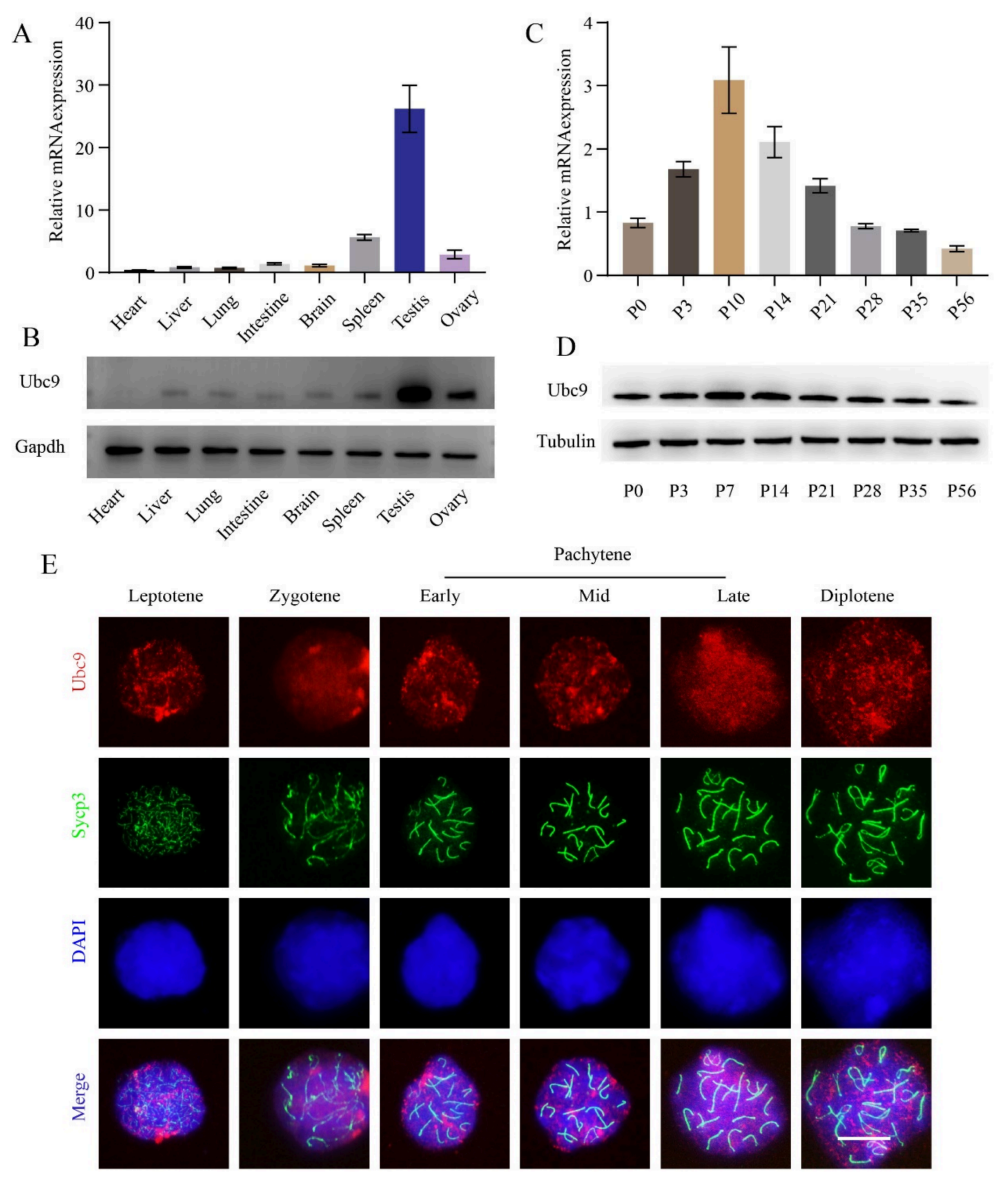
The dynamic expression pattern of Ubc9 in mice. (**A**) Expression of Ubc9 mRNA in multiple organs of adult wild-type mice examined via qRT-PCR. (**B**) Levels of Ubc9 protein in multiple organs of adult wild-type mice via Western blotting. (**C**) qRT-PCR analysis of mouse testes on different postnatal days. (**D**) The results of Western blotting of mouse testes on various postnatal days. (**E**) The dynamic expression pattern of Ubc9 during meiosis I was detected via a meiotic chromosome spread assay. Scale bars = 5 μm. Data are presented as mean ± SD, *n* = 3; a *t*-test was performed. The uncropped blots are shown in Figure S3.

To investigate the role of Ubc9 in mammals, we first detected the expression profile of Ubc9 in mice. The qRT-PCR and Western blotting of various organs revealed higher mRNA and protein expression levels of Ubc9 in adult testes (Figure 1A,B). The qRT-PCR and Western blotting of the developmental stages of testes showed that the mRNA and protein expression levels of Ubc9 were higher on postnatal days 7 and 14 than on other days (Figure 1C,D). In addition, we used the Male Health Atlas database (http://malehealthatlas.cn/) to further analyze expression levels of Ubc9 in single germ cells [28]. The results revealed that Ubc9 was expressed throughout the entire process of spermatogenesis, with slightly higher expression at the zygotene stage compared to other periods of germ cells (Supplementary Figure S1A). This finding indicates the potential involvement of Ubc9 in early meiosis. Sycp3, an important component of the synaptonemal complex, serves as a crucial marker during meiosis I. Using a chromosome spread assay to detect the subcellular location of Ubc9 in spermatocytes, we found that Ubc9 was diffusely distributed within spermatocytes, partially co-localizing with the synaptonemal complex (Figure 1E).

### 3.2. Ubc9 Is Essential for Spermatogenesis and Male Fertility

**Figure 2:**
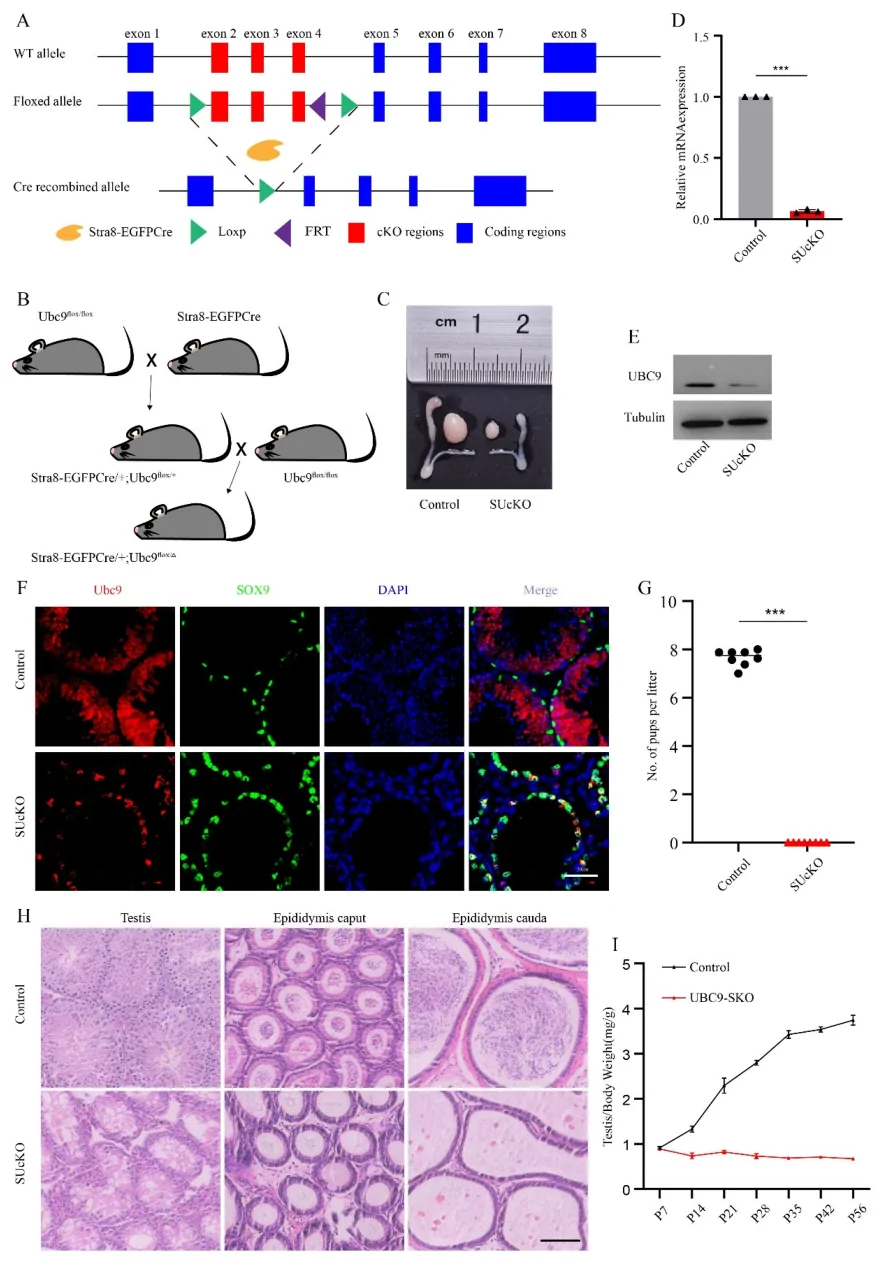
Ubc9 is required for spermatogenesis and male fertility. (**A**) Schematic diagram representing a floxed Ubc9 allele to generate a knockout allele via homologous recombination with the cyclization recombination enzyme (Stra8-EGFPCre). (**B**) Schematic diagram representing the generation of a conditional Ubc9 knockout mouse with Stra8-EGFPCre. (**C**) Gross morphology of the testes and the epididymides from adult control and SUcKO mice. (**D**) The mRNA expression of Ubc9 in control and SUcKO mouse testes examined via RT-PCR. Data are presented as mean ± SD, *n* = 3; a *t*-test was performed. *** *p* < 0.001. (**E**) Levels of Ubc9 protein in control and SUcKO mouse testes at postnatal day 12. (**F**) Immunofluorescence staining of Ubc9 and SOX9 was performed in adult control and SUcKO mouse testes. Scale bars = 50 μm. Data are presented as mean ± SD, *n* = 3; a *t*-test was performed. (**G**) The number of pups per litter from adult control and SUcKO mice. Data are presented as mean ± SD, *n* = 8; a *t*-test was performed. *** *p* < 0.001 (**H**) HE staining of testes and epididymides from control and SUcKO mice at postnatal day 56. Scale bars = 50 μm. (**I**) The testis/body weight (mg/g) of control and SUcKO mice on different postnatal days is shown. The uncropped blots are shown in Figure S3.

To further explore the functions of Ubc9 during meiosis, we acquired gene-edited mice with a floxed-Ubc9 allele on chromosome 17 (Figure 2A). By crossing floxed-Ubc9 mice with Stra8-GFPCre mice, we obtained the germ cell-specific conditional Ubc9 knockout mice (Figure 2B). Stra8 mainly exerts effects during differentiated spermatogonia, until the initiation of meiosis. Genotype identification was performed via PCR and 2.5% agarose gel electrophoresis (Supplementary Figure S2A–C). The testes of SUcKO are much smaller than those of their control siblings (Figure 2C). We used qRT–PCR and Western blotting to detect the efficiency of Ubc9 deletion. The results revealed that the expression of Ubc9 was significantly lower in SUcKO mice compared with the control group at postnatal day 16 (Figure 2D,E). SOX9 is an important marker in Sertoli cells. Moreover, immunofluorescence staining revealed that the expression of Ubc9 in germ cells from SUcKO mice disappeared compared with control mice; however, the level of Ubc9 remained unchanged in somatic cells between the two groups (Figure 2F). Despite the SUcKO mice appearing normal and healthy, they were found to be infertile in the 6-month fertility test (Figure 2G). HE staining of the testes and epididymides revealed a marked loss of germ cells in the seminiferous tubules of the SUcKO group compared with the control group. Moreover, sperm were completely absent from both the caput and cauda of the epididymis in the SUcKO group (Figure 2H). Subsequently, the ratio of testis weight to body weight revealed a significant difference between postnatal days 7 and 14 (Figure 2I). Taken together, these results suggest that Ubc9 may play a key role in early meiosis.

### 3.3. Loss of Ubc9 Results in Meiosis Stagnation

**Figure 3:**
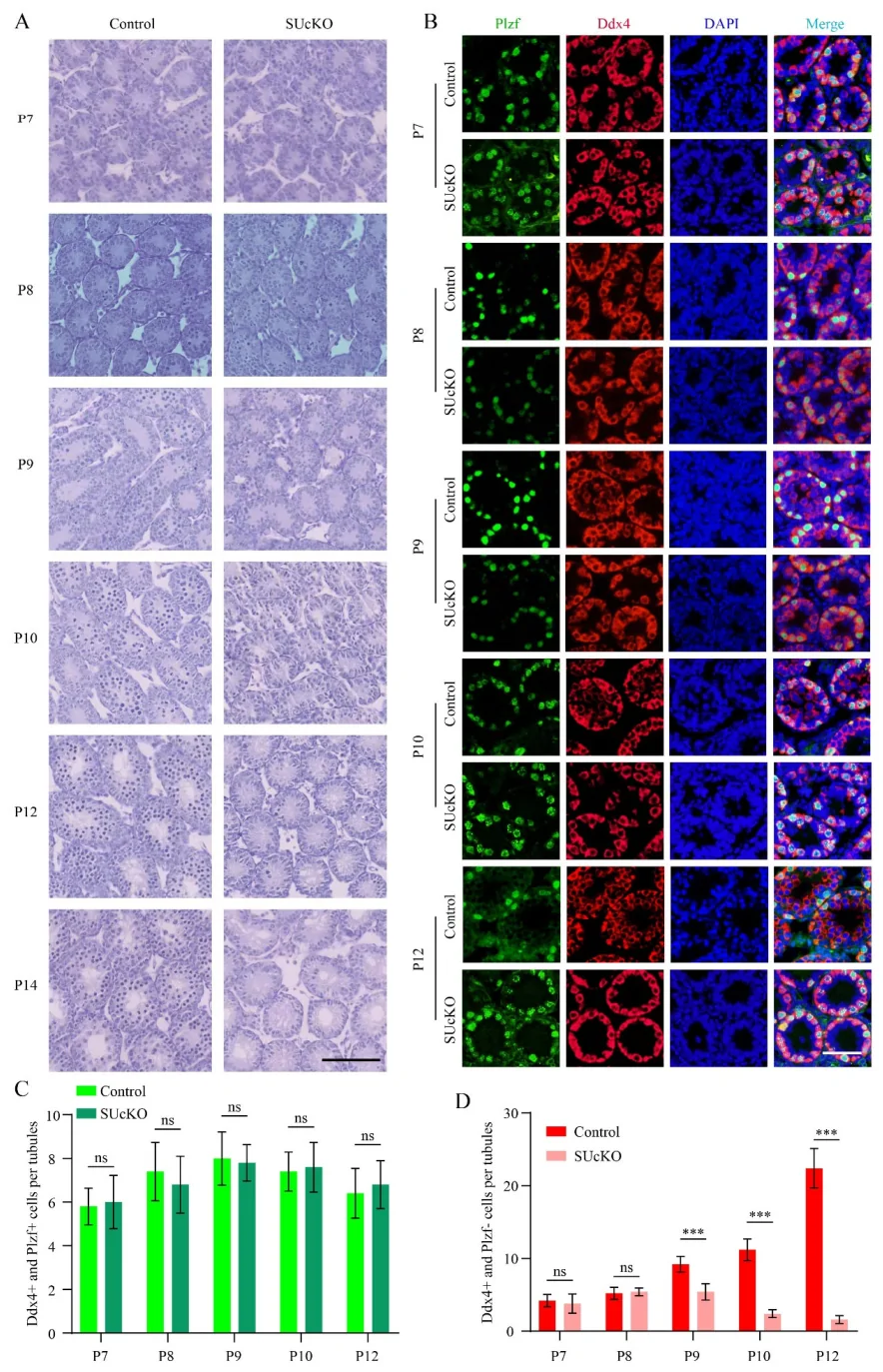
Conditional Ubc9 knockout causes the loss of germ cells. (**A**) PAS staining of control and SUcKO mice on different postnatal days. Scale bars = 50 μm. (**B**) Immunofluorescence staining of Ubc9 and SOX9 was performed in control and SUcKO mice on different postnatal days. Scale bars = 20 μm. (**C**) Analysis of Ddx4-positive and Plzf-positive cells per tubule. Data are presented as mean ± SD; a *t*-test was performed. (**D**) The analysis of Ddx4-positive but Plzf-negative cells per tubule. Data are presented as mean ± SD; a *t*-test was performed. *** *p* < 0.001.

Due to Ubc9 deficiency causing abnormal testes before postnatal day 14, we further explored the changes in meiosis. We firstly performed PAS staining and discovered that the number of germ cells had significantly decreased at postnatal day 9 compared with the control group (Figure 3A). Plzf is a marker of undifferentiated spermatogonia. In adult testes, DDX4 is a predominant marker in spermatogonia, spermatocytes, and round spermatids, with the signal mainly localized in the cytoplasm. Thereafter, we performed immunofluorescence staining with Plzf and Ddx4 to observe the changes in germ cells (Figure 3B). The results revealed that the Plzf-positive cells in the SUcKO mice testis at postnatal days 7, 8, 9, 10, and 12 did not exhibit significant differences compared to the control group (Figure 3C). However, the number of Ddx4-positive but Plzf-negative germ cells was significantly reduced at postnatal day 9 in the SUcKO group compared with the control group (Figure 3D). Therefore, we speculate that the initiation of meiosis occurred abnormally in Ubc9-defective mice.

**Figure 4:**
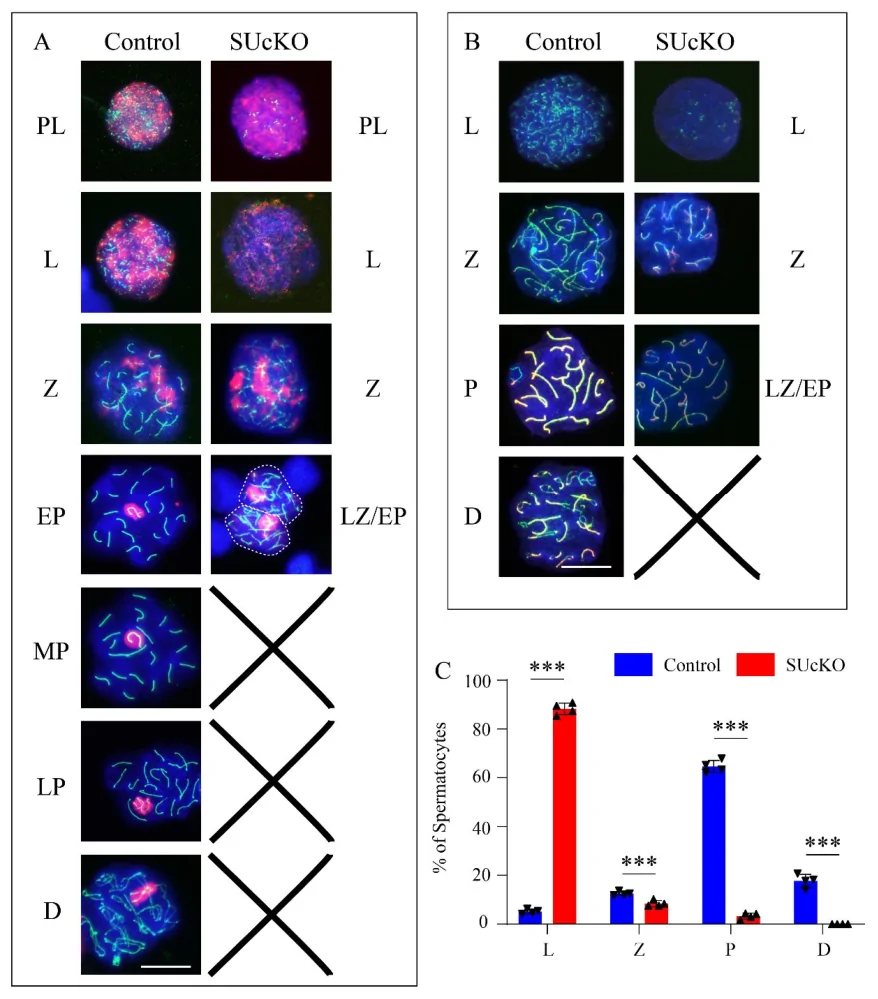
Loss of Ubc9 caused meiotic arrest. (**A**) Immunofluorescence staining with Sycp3 and γ-H2AX on spermatocytes from control and SUcKO mice. Scale bars = 5 μm. (**B**) Immunofluorescence staining with Sycp1 and Sycp3 on spermatocytes from control and SUcKO mice. Scale bars = 5 μm. (**C**) The percentage of spermatocytes at different stages is shown. Data are presented as mean ± SD, *n* = 4; a *t*-test was performed. PL: preleptotene; L: leptotene; Z: zygotene; LZ: late zygotene; P: pachytene; EP: early pachytene; MP: mid pachytene; LP: late pachytene; D: diplotene. × indicates no corresponding spermatocytes. *** *p* < 0.001.

To better understand the abnormalities in meiosis, we performed a meiotic chromosome spread assay. We used γ-H2AX as a marker due to its specific expression and localization patterns during different stages of meiosis I. Sycp3 is a marker of synapsis, located on the chromosome. Our results revealed that meiosis was completely arrested at the late zygotene stage and early pachytene stage (LZ/EP), with severe abnormalities of the synaptonemal complex in the SUcKO group (Figure 4A). Moreover, in the leptotene stage, the fluorescence signal of Sycp3 was significantly shorter and reduced; however, the fluorescence signal of γ-H2AX was normal in the SUcKO group compared with the control group, suggesting that the initiation of the synaptonemal complex was abnormal. Sycp1 is a component of the central element in the synaptonemal complex. Consequently, the results of immunofluorescence staining with Sycp1 revealed that the expression of Sycp1 was markedly reduced in LZ/EP stages and significantly defective on the axes of the synaptonemal complex in the SUcKO group compared with the control group (Figure 4B). In addition, we counted the number of different spermatocytes during meiosis I. In the SUcKO group, compared to the control group, the results showed that the number of spermatocytes in the leptotene stage increased abnormally by more than 90%, with only a few germ cells developing into the zygotene stage and an extremely small number progressing to the early pachytene stage, while diplotene cells were completely absent (Figure 4C). Taken together, these results showed that the deletion of Ubc9 caused abnormal meiosis initiation.

### 3.4. Absence of Ubc9 Disrupts SUMO Modification

**Figure 5:**
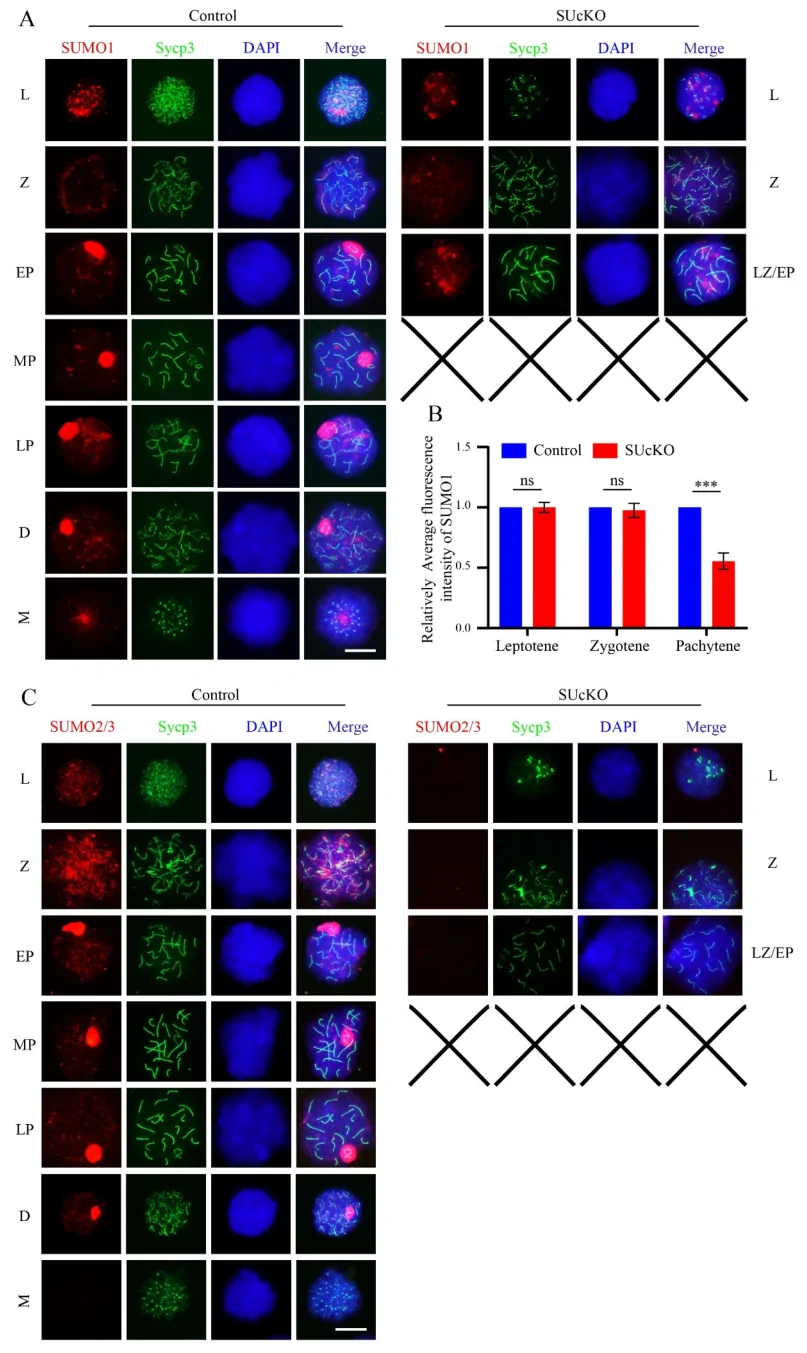
Ubc9 deficiency altered SUMO modification. (**A**) Immunofluorescence staining with Sycp3 and SUMO1 on spermatocytes from control and SUcKO mice. Scale bars = 5 μm. (**B**) The fluorescence intensity of SUMO1 in spermatocytes at different stages. Data are presented as mean ± SD; a *t*-test was performed. *** *p* < 0.001. (**C**) Immunofluorescence staining with Sycp3 and SUMO2/3 on spermatocytes from control and SUcKO mice. Scale bars = 5 μm. L: leptotene; Z: zygotene; LZ: late zygotene; P: pachytene; EP: early pachytene; MP: mid pachytene; LP: late pachytene; D: diplotene. M: metaphase. × indicates no corresponding spermatocytes.

SUMO proteins, which serve as key modifiers in Ubc9-mediated SUMOylation, are classified into five subtypes. Given their widespread distribution across various organs, SUMO1 and SUMO2/3 warrant further investigation to determine their roles in Ubc9-deficient spermatocytes [29]. We performed a meiotic chromosome spread assay to observe the distribution of SUMO1 and SUMO2/3 in the nucleus and the changes after Ubc9 deletion. The results showed that SUMO1 exhibited a dot-like random distribution in the nucleus during the leptotene stage in the control group. The distribution during the zygotene stage was more dispersed, with dot-like distributions in the nucleus, most of which co-localize with the chromosome axes in the control group. In the pachytene and diplotene stages, they mainly concentrate on the XY body, while other parts exhibit a dot-like distribution in the nucleus, with a small portion co-localizing with the chromosome axes in the control group. During metaphase, SUMO1 was concentrated in a specific area in the control group (Figure 5A). The absence of Ubc9 did not affect SUMO1 in the leptotene- and zygotene-stage spermatocytes, with no significant difference in average fluorescence intensity in the SUcKO group compared with the control group. However, the absence of Ubc9 resulted in a failure of SUMO1 to accumulate on the XY body in LZ/EP stages, with a significant decrease in average fluorescence intensity in the SUcKO group compared with the control group (Figure 5B).

In the control group, SUMO2/3 shows a dot-like distribution during meiosis at the leptotene stage, with most signals co-localizing with the chromosome axes. The fluorescence intensity of SUMO2/3 significantly increased at the zygotene stage, with some co-localizing with Sycp3 green fluorescence signals in the control group. In the control group at the pachytene and diplotene stages, SUMO2/3 fluorescence signals are mainly concentrated on the XY body, while some fluorescent signals are distributed in a punctate manner in other regions, with some co-localizing with the chromosome axes. During the metaphase of meiosis, the fluorescent signals completely disappear (Figure 5C). Loss of Ubc9 resulted in the complete disappearance of SUMO2/3 fluorescent signals during the zygotene and pachytene stages in the SUcKO group compared with the control group (Figure 5C). Combined with the aforementioned results, we posit that SUMO2/3 may play a more crucial role in meiosis than SUMO1 and that Ubc9 mainly regulates the initiation of meiosis via SUMO2/3 rather than SUMO1.

### 3.5. Ubc9 Regulates Gene Expression Related to the Formation of the Synaptonemal Complex

**Figure 6:**
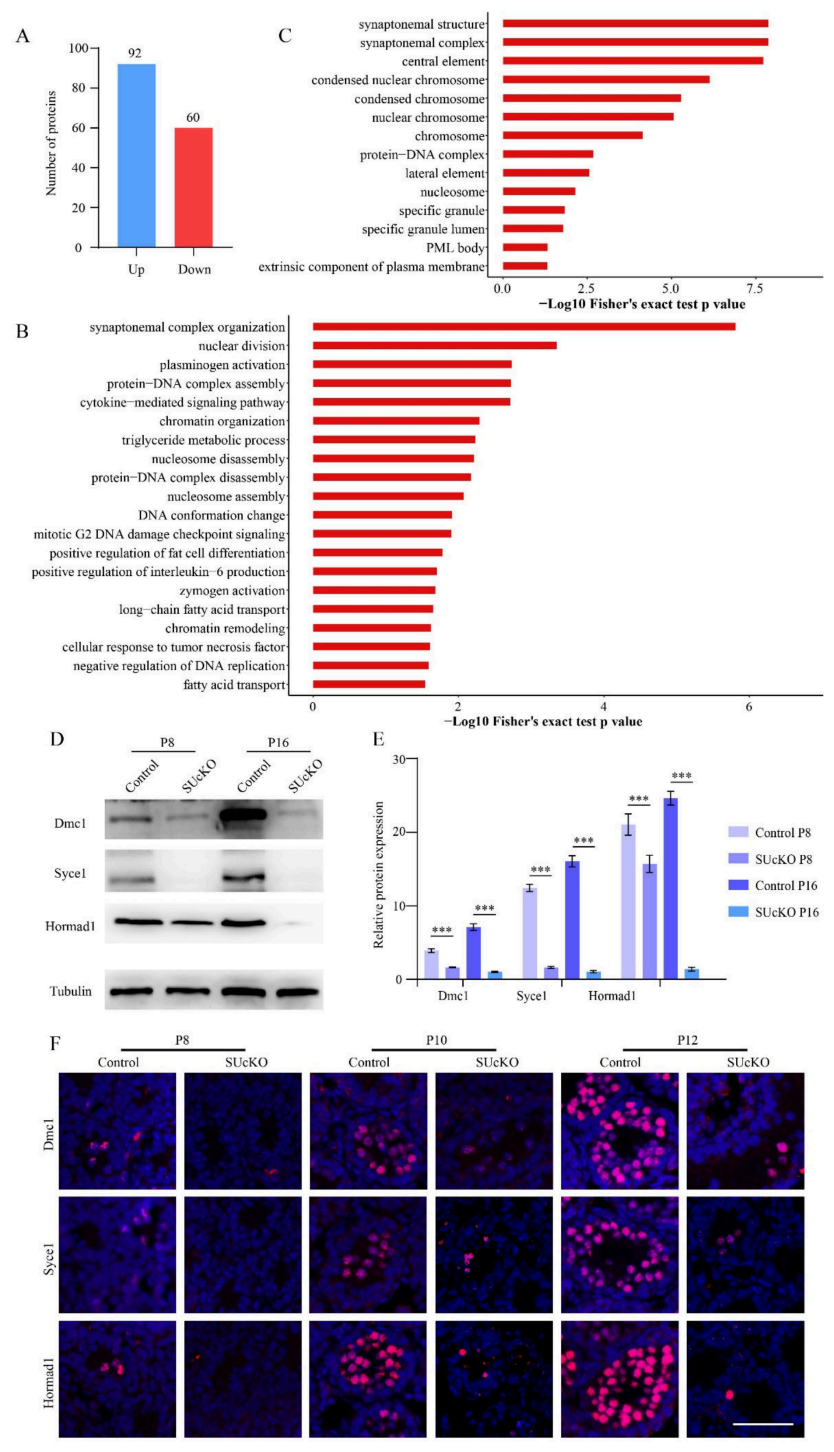
Ubc9 influenced the expression of proteins related to the synaptonemal complex. (**A**) The number of upregulated and downregulated genes in SUcKO mice compared with control mice. (**B**) The GO analysis of biological processes in control and SUcKO mice performed via proteomics. (**C**) The GO analysis of cellular components in control and SUcKO mice performed via proteomics. (**D**) Western blotting of significantly changed proteins in control and SUcKO mice at postnatal days 8 and 16. (**E**) Quantitative analysis of relative protein expression. *** *p* < 0.001. (**F**) Immunofluorescence staining with Syce1, Hormad1, and Dmc1 in control and SUcKO mice at postnatal days 8, 10, and 12. Scale bars = 20 μm. The uncropped blots are shown in Figure S3.

To further explore the effects of Ubc9 during meiosis, we collected testicular tissue from postnatal day 8 mice for proteomic analysis. We identified a total of 96 and 60 genes as upregulated and downregulated, respectively, in SUcKO mice compared with control mice (Figure 6A). We further performed GO analysis, with our results revealing that the downregulated genes were related to our observation. The results revealed that the enrichment of biological processes mainly focused on synaptonemal complex organization, nuclear division, and plasminogen activation (Figure 6B). The enrichment of cellular components mainly focused on synaptonemal structure, synaptonemal complex, and central element (Figure 6C). We further analyzed the results of the enrichment of cellular components, with our results revealing five downregulated proteins, including Syce1, Hormad1, Sycp1, Tex12, and Syce2. In addition to the five aforementioned proteins, we detected DMC1 along the condensed nuclear chromosome, which plays a key role in meiosis. We selected Dmc1, Syce1, and Hormad1 for verification. Our Western blotting results revealed that the expression levels of Dmc1, Syce1, and Hormad1 significantly decreased in postnatal day 8 mice of the SUcKO group compared with the control group. In postnatal day 16 mice, the expression levels of Dmc1, Syce1, and Hormad1 were markedly lower in the SUcKO group compared with the control group (Figure 6D,E). In addition, we performed immunofluorescence staining with Dmc1, Syce1, and Hormad1 in postnatal day 8, 10, and 12 mice. The expression levels of Dmc1, Syce1, and Hormad1 gradually increased with the development of testes in the control group; however, their expression remained at a lower level in the SUcKO group (Figure 6F). Our findings indicated that Ubc9 regulated the protein levels involved in the formation of the synaptonemal complex.

## 4. Discussion

Spermatogenesis is a complex and highly ordered process involving DNA replication and transcription. Chromatin is continuously fixed to the axes of the synaptonemal complex, enabling pairing and crossing over between homologous chromosomes [30]. The formation of the synaptonemal complex, which is regulated by gene transcription, translation, and post-translational modifications, is crucial for meiosis and the generation of haploid gametes [31]. For example, hnRNPK regulates the process of meiosis by affecting alternative splicing of precursor mRNAs related to meiosis [32]. In this study, we explored the expression pattern of Ubc9 in germ cells, which is mainly highly expressed in early meiosis. By constructing a differentiated spermatogonia cell-specific Ubc9 knockout mouse model, we revealed that the loss of Ubc9 resulted in male infertility, testicular atrophy, and a substantial loss of germ cells in the seminiferous tubules. We found that Ubc9 influenced post-translational modifications through SUMO2/3 during meiosis. Moreover, conditional loss of Ubc9 with Gdf9-Cre in female mice resulted in stagnation of follicular development, meiotic defects, abnormal communication between oocytes and granulosa cells, and female infertility [33], indicating that Ubc9 is critical for the development of both male and female germ cells.

Ubc9-dependent SUMOylation is involved in the regulation of gene expression, affecting not only the binding between transcription factors and promoters but also various translation-related proteins during ribosome assembly and export [34,35]. We found that many spermatocytes were in a malformed leptotene stage in SUcKO mice. We hypothesized that this phenomenon was caused by the decreased expression levels of proteins related to the formation of the synaptonemal complex, which was due to the loss of SUMOylation; the proteomic analysis confirmed this. Our analysis revealed that the expression of several key proteins involved in the formation of the synaptonemal complex, including Syce1, Syce2, Hormad1, Tex12, and Sycp1, was significantly decreased. Consequently, loss of Ubc9 resulted in abnormalities during the initiation and elongation of the synaptonemal complex. Although a very small number of spermatocytes progressed to the late zygotene or early pachytene stages, the reduction in Dmc1 further caused abnormalities in the recognition, recombination, and crossover between homologous chromosomes. The absence of Ubc9 led to abnormalities in multiple factors during spermatogenesis, ultimately resulting in a complete arrest of germ cell meiosis and the failure of haploid gamete generation.

In addition, we explored the impact of Ubc9 deficiency on various SUMO molecules during different stages of meiosis I. The fluorescence signals of SUMO2/3 molecules essentially disappeared in spermatocytes at the leptotene stage, preventing them from participating in biological activities at the onset of meiosis. We speculated that SUMO2/3 is a critical mediator for Ubc9 to exhibit biological functions in meiosis. In contrast, the fluorescence intensity of SUMO1 molecules did not exhibit significant changes in spermatocytes at the leptotene and zygotene stages between the control group and the SUcKO group and only decreased when the spermatocytes completely arrested at the early pachytene stage in the SUcKO group. Moreover, SUMO1 remained at the metaphase stage in the control group, while the fluorescence signals of SUMO2/3 completely disappeared at this point. We speculated that SUMO1 may play a weaker regulatory role in meiosis but may exert effects following meiosis. In addition, research has demonstrated that SUMO1 is highly expressed in the acrosome of buffalo sperm, suggesting its involvement in dissolving the zona pellucida surrounding the follicle and promoting oocyte fertilization [36]. Furthermore, SUMO1-mediated SUMOylation has been closely linked to sperm vitality and motility in human defective sperm [37]. However, regrettably, our conditional knockout model has already undergone arrest during meiosis, making it impossible to subsequently observe the regulatory effects of Ubc9 on spermiogenesis. In future studies, researchers could utilize other Cre mice (such as Prm-Cre) to further explore the impact of Ubc9 on spermiogenesis or sperm vitality and motility.

During the pachytene and diplotene stages, most chromatin of X and Y chromosomes does not undergo pairing due to the short homologous region (pseudo-autosomal region) on the XY body [38]. The stability and dynamics of the XY body require the participation of various proteins, such as TOPBP1, BRCA1, G6PC3, and LARP7, which affect the separation of sex chromosomes [39,40,41]. In our study, we did not observe the synapsis between sex chromosomes after the deletion of Ubc9. One possibility is that the developmental arrest of the spermatocytes occurs before the synapsis of sex chromosomes; another explanation could be that specific SUMO-modified proteins are required for the occurrence and maintenance of the synapsis of sex chromosomes. In our study, we found that SUMO1 and SUMO2/3 significantly accumulated at the XY body, while both failed to aggregate at the XY body after the deletion of Ubc9. Hence, we speculated that Ubc9-mediated SUMO1 and SUMO2/3 modifications may play a crucial role in the formation and maintenance of the XY body. Future studies involving single-cell sequencing or single-cell proteomics may aid in uncovering key regulatory molecules for the formation and dynamic changes of the XY body in Ubc9-deficient spermatocytes at the pachytene stage.

## 5. Conclusions

In summary, we identified Ubc9 as a critical regulator of male fertility and the formation of the synaptonemal complex. Conditional Ubc9 knockout mice exhibited complete male infertility, testicular atrophy, and a severe deficiency of germ cells. The initiation and maintenance of the synaptonemal complex were disrupted due to abnormalities in crucial proteins related to meiosis. Importantly, we revealed that Ubc9 regulates the process of meiosis via SUMO2/3 rather than SUMO1. In the future, more gene knockout mouse models may provide us with more information to investigate the causes of male infertility and open up new avenues for in vitro germ cell culture and development.

## Data Availability

This article and Supplementary Materials files include sufficient data to verify our findings. Requests for access to the datasets or other data used in this study can be directed to the corresponding author.

## Supplementary Materials

The following supporting information can be downloaded at: https://www.mdpi.com/article/10.3390/biomedicines14092129/s1, Figure S1. The expression of Ubc9 in single cell database. A: The data and figure showed the expression of Ubc9 in different germ cells, which derived from Male Health Atlas. Figure S2. Genotyping identification of gene-edited mice. A: Genotyping images of tails from Stra8-EGFPCre and wild-type mice. B: Genotyping images of tails from different mice, including flox/Del (or flox/flox), flox/+, and wild-type. C: Genotyping images of tails from flox/Del and wild-type mice. Figure S3. The full Blots image. Table S1. Primer information in this study. Table S2. information of reagents in immunofluorescence and western blot.

## Abbreviations

Ddx4	DEAD-box helicase 4
Dmc1	DNA meiotic recombinase 1
Hormad1	HORMA domain containing 1
Plzf	promyelocytic leukemia zinc finger
Sox9	SRY-box transcription factor 9
SUMO1	small ubiquitin-like modifier 1
SUMO2/3	small ubiquitin-like modifier 2/3
Syce1	synaptonemal complex central element protein 1
Syce2	synaptonemal complex central element protein 2
Sycp1	synaptonemal complex protein 1
Sycp3	synaptonemal complex protein 3
Tex12	testis expressed 12
Ubc9	ubiquitin carrier protein 9
