# Supplementary material for: Ubc9 Mediates SUMO2/3 Modification to Regulate Meiosis in Male Mice
Source: Biomedicines. 2026 Sep 21;14(9):2129. doi: 10.3390/biomedicines14092129 (PMC13604139; doi:10.3390/biomedicines14092129)
Supplement: Supplementary file 1 [file biomedicines-14-02129-s001.zip › figures.pdf]

Supplementary information

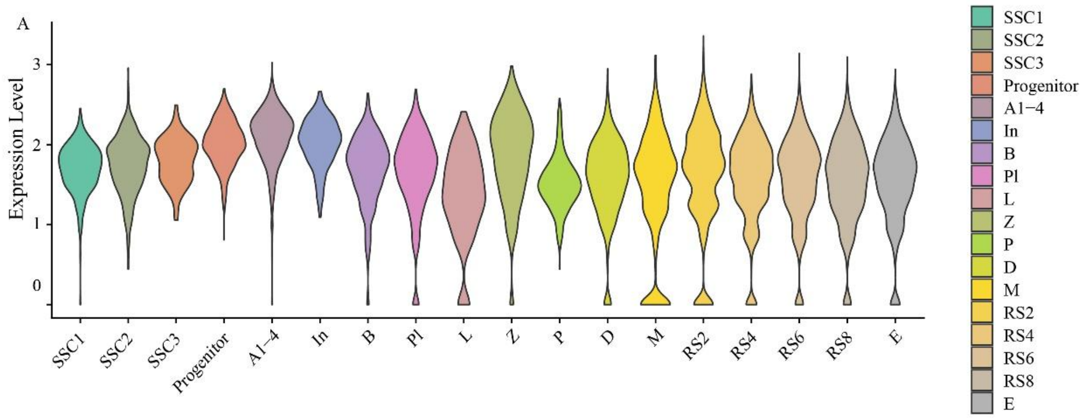

Figure S1. The expression of Ubc9 in single cell database. A: The data and figure showed the expression of Ubc9 in different germ cells, which derived from Male Health Atlas.

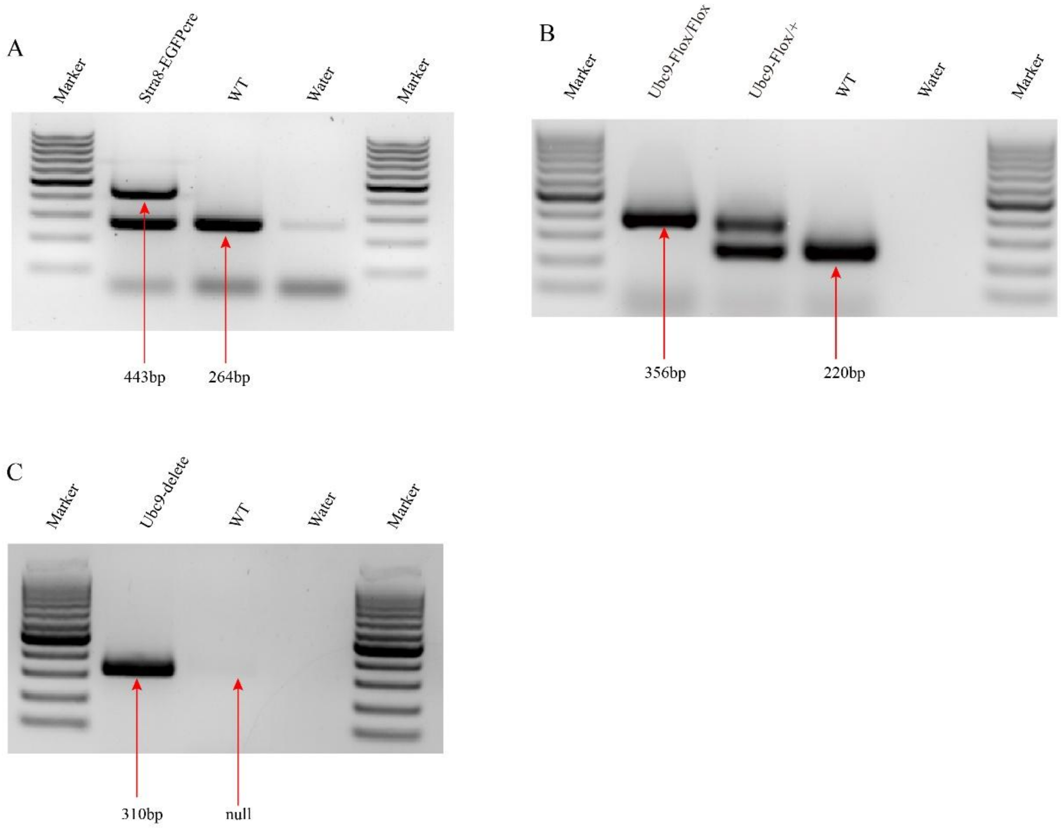

Figure S2. Genotyping identification of gene-edited mice. **A**: Genotyping images of tails from Stra8-EGFPCre and wild-type mice. **B**: Genotyping images of tails from different mice, including flox/Del (or flox/flox), flox/+, and wild-type. **C**: Genotyping images of tails from flox/Del and wild-type mice.

Figure 1C

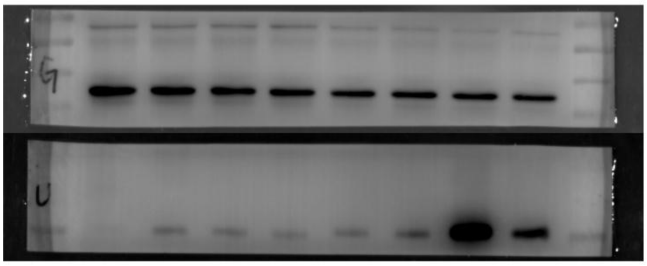

Figure 1D

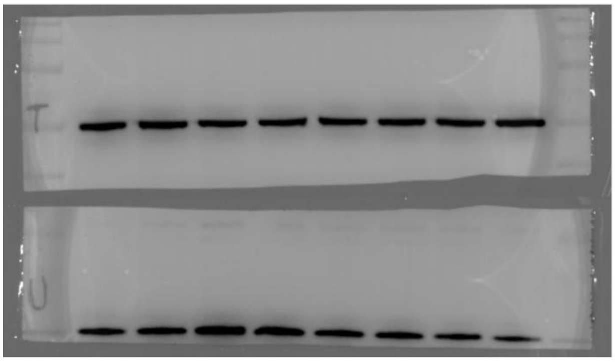

Figure 2E

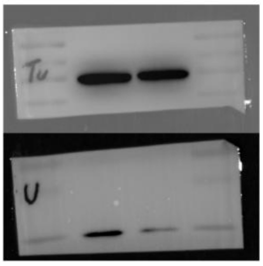

Figure 6D

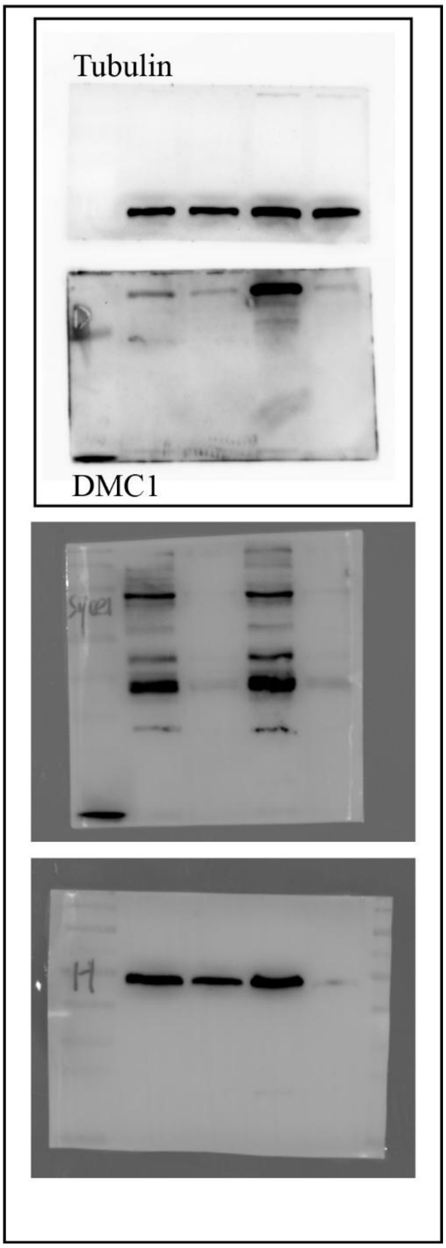

Figure S3. The full Blots image.
