# Supplementary material for: Ubc9 Mediates SUMO2/3 Modification to Regulate Meiosis in Male Mice
Source: Biomedicines. 2026 Sep 21;14(9):2129. doi: 10.3390/biomedicines14092129 (PMC13604139; doi:10.3390/biomedicines14092129)
Supplement: Supplementary file 1 [file biomedicines-14-02129-s001.zip › table.pdf]

Table S1. Primer information in this study.

Gene Name	Sequence (5'-3')	Application
Gapdh	F: AGGTCGGTGTGAACGGATTTG	RT-PCR
	R: GGGGTCGTGATGGCAACA	
Tubulin	F: TACACCAACCTTAACCGCCT	RT-PCR
	R: GCTGCTCATGGTAGGCTTTC	
Ubc9	F1: ATGAACTGGGAGTGCGCTAT	RT-PCR
	R1: CATTTTGGTGGTGAGGACGG	
	F2: ATGGGAAGGAGGCTTGTTCA	
	R2: TTGATGGTGATAGCTGGCCT	
Stra8-EGFPCre	F1: GATGGATTTCCGTCTCTGGTGTAG	Genotyping
	R1: CCCATTTAATCTCCTCCTTCTCCG	
	F2: GTCAGAGAAGGTTGTATCGAACTGG	
Ubc9-flox	F: GCCTGACCTGGATCTTGCCCTC	Genotyping
	R: TCTGAGGCTAAGATGTATGGCCAGCT	
Ubc9-delete	F: ACCTGGATCTTGCCCTCCTT	Genotyping
	R: CAGCTCAGACCTGGCCTTACT	

Table S2. information of reagents in immunofluorescence and western blot.

Gene Name	Cat NO.	Application (Concentration)	Company
DDX4	Ab284611	IF（1：400）	Abcam
PLZF	sc-28319	IF（1：100）	Santa
SOX9	A19710	IF（1：200）	ABclonal
UBC9	10070-1-AP	WB（1：1000）	Proteintech
UBC9	DF6916	IF（1：100）	Affinity
SYCP3	ab97672	IF（1：500）	Abcam
SYCP1	ab15090	IF（1：500）	Abcam
γ-H2AX	ab11174	IF（1：2000）	Abcam
SUMO1	PTM6055	IF（1：100）	PTM BIO
SUMO2/3	11251-1-AP	IF（1：100）	Proteintech
GAPDH	ABL1020	WB（1：10000）	Abbkine
Tubulin	66031-1-Ig	WB（1：20000）	Proteintech
HRP-conjugated Goat anti-Rabbit IgG	AS014	WB（1：500）	Abclonal
HRP-conjugated Goat anti-Mouse IgG	AS003	WB（1：500）	Abclonal
Coralite488-conjugated Goat Anti-Mouse IgG	SA00013-1	IF（1：500）	Proteintech
HORMAD1	67091-1-Ig	WB（1：1000）<br>IF（1：100）	Proteintech
SYCE1	11063-1-AP	WB（1：1000）<br>IF（1：100）	Proteintech
DMC1	13714-1-AP	WB（1：1000）<br>IF（1：100）	Proteintech
Coralite594-conjugated Goat Anti-Rabbit IgG	SA00013-4	IF（1：500）	Proteintech

WB: Western blot; IF: Immunofluorescence
